# Anoctamin 6 differs from VRAC and VSOAC but is involved in apoptosis and supports volume regulation in the presence of Ca^2+^

**DOI:** 10.1007/s00424-013-1428-4

**Published:** 2014-01-14

**Authors:** C. A. Juul, S. Grubb, K. A. Poulsen, T. Kyed, N. Hashem, I. H. Lambert, E. H. Larsen, E. K. Hoffmann

**Affiliations:** Department of Biology, August Krogh Building, 13 Universitetsparken, 2100 Copenhagen Ø, Denmark

**Keywords:** ANO6, TMEM16F, VRAC, Volume regulation, Apoptosis

## Abstract

Anoctamin 6 (ANO6), also known as TMEM16F, has been shown to be a calcium-activated anion channel with delayed calcium activation. The cellular function of ANO6 is under debate, and different groups have come to different conclusions about ANO6’s physiological role. Although it is now quite well established that ANO6 is distinct from the volume-regulated anion channel, it is still unclear whether ANO6 or other anoctamins can be activated by cell swelling. In this study, we suggest that ANO1, ANO6, and ANO10 do not contribute to the volume-activated current in ANO-overexpressing HEK293 cells. Furthermore, knock-down of ANO6 in Ehrlich ascites tumor cells (EATC) and Ehrlich–Lettre ascites (ELA) did not decrease but instead significantly increased swelling-activated membrane currents. Knock-down of ANO6 in EATC did not reduce regulatory volume decrease (RVD) in the absence of extracellular calcium, whereas it significantly reduced RVD in the presence of calcium. Interestingly, we found that knock-down of ANO6 in ELA cells resulted in a decrease in cisplatin-induced caspase-3 activity, confirming earlier findings that ANO6 is involved in apoptosis. Finally, knock-down of ANO1 and ANO6 did not affect the volume-sensitive release of taurine in ELA cells. Thus, our data provide evidence that ANO6 cannot be activated directly by cell swelling unless Ca^2+^ is present. We also conclude that ANO6 carries a current during RVD, provided extracellular calcium is present. Thus, swelling activation of ANO6 requires the presence of free calcium.

## Introduction

Anoctamin 6 (ANO6), also known as TMEM16F, is a member of the anoctamin family of transmembrane proteins, which comprises ten members in vertebrates (ANO1–10). Three of the members, ANO1, ANO2, and ANO6 have convincingly been shown to be calcium-activated chloride channels (CaCCs) [2, 9, 28, 31–33, 36], whereas the functions of the remaining members are less well known. Several anoctamins are known to be mutated or dysregulated in human diseases, such as musculoskeletal disorders, ataxias, and several types of cancer, which emphasizes the need to thoroughly study them [4]. ANO6 is interesting because it has been shown that mutations in this protein are the cause of the congenital bleeding disorder Scott syndrome in humans [34]. ANO6’s involvement in blood coagulation was confirmed in a study of ANO6 knock-out mice, which showed prolonged bleeding time compared with wild-type (WT) mice [37]. Another knock-out mouse study suggested that ANO6 is also important for embryonic development of skeletal tissues [6]. Finally, changes in alternative splicing of human ANO6 are associated with the development of metastasis in breast cancer [5]. ANO6 has been suggested to be involved in a diverse array of processes in the cell, such as externalization of phosphatidylserine [23, 34], cell volume regulation [1], and cell migration [13]. Although it is now well established that ANO6 constitutes a Ca^2+^- and depolarization-activated anion channel [1, 24, 31, 33], Yang et al. found that it was a nonselective cation channel [37]. Our group recently conducted a thorough investigation of the properties of the ANO6 channel [9]. We found that ANO6 is a plasma membrane-located Ca^2+^-activated anion channel with a relatively high cation permeability that is correlated with the ANO6-associated anion permeability (P_Na_ = 0.3 P_Cl_). ANO6’s properties differed significantly from those of ANO1, including a markedly higher EC_50_ for Ca^2+^ and a delay of several minutes from cytosolic (Ca^2+^) increase to Cl^−^ current activation.

In a study from Almaca et al., it was suggested that some anoctamins, including ANO6, contribute to the swelling-activated chloride current (*I*_Cl, swell_) [1]. Knock-down (KD) of ANO6 significantly reduced *I*_Cl, swell_ and impaired the cell’s ability to undergo regulatory volume decrease (RVD) in response to osmotic cell swelling. Recently, however, the results of Shimizu et al. showed that ANO6 is different from the volume-regulated anion channel (VRAC) [33]. Furthermore, Kunzelmann et al. excluded ANO6 as a VRAC candidate, as ANO6 has none of the characteristic fingerprints of VRAC, i.e., isovolumic activation by a decrease in intracellular ionic strength or cell perfusion with GTPγS [17]. Finally, our studies indicated a significant difference between the two channels as revealed by their current responses to a step voltage protocol. Following the initial ohmic current response to a V-step into the positive region of membrane potentials, ANO6 currents are slowly activated [9, 25] in contrast to VRAC currents which are known to be slowly deactivated [12, 24]. ANO6 is thus not identical with VRAC. However, there are still ongoing discussions as to whether ANO6 is activated by cell swelling and whether ANO6 contributes to RVD. This is investigated in the present study. Additionally, we looked at the relation between ANO6 and another still unidentified anion channel, the volume-sensitive organic osmolyte and anion channel (VSOAC), which has previously been suggested to be identical to VRAC, although convincing evidence now indicate that they are distinct channels [12]. Finally, we examined the possible role of ANO6 in cisplatin-induced apoptosis, which is a process that is highly dependent on cell shrinkage [29].

## Materials and methods

### Cell culture and transfection

Three different model cell lines were used: The mouse-derived Ehrlich ascites tumor cells (EATC) and Ehrlich–Lettre ascites (ELA) cells and the human-derived human embryonic kidney 293 (HEK293) cells. All cells were maintained at 37 °C aerated with 5 % CO_2_:95 % O_2_. HEK293 cells were maintained in DMEM and EATC and ELA cells were kept in RPMI-1640. 10 % fetal bovine serum and 1 % penicillin/streptomycin were added to each media. The medium for the EATC and ELA KD clones and the EATC overexpression clones was supplemented with 10 μg/mL blasticidin to select for resistant clones. EATC and ELA cells were transfected using Lipofectamine 2000 (Invitrogen, Taastrup, Denmark) and HEK293 cells using Trans-IT®-293 (Mirus Bio LLC, Madison, WI, USA). HEK293 cells were used for electrophysiological recordings 24–48 h after transfection.

### Isolation of RNA, reverse transcription, PCR, and cDNA cloning

Total RNA was isolated from EATC using the RNeasy Mini Kit (Qiagen, Ballerup, Denmark). cDNA was prepared in a total volume of 40 μL by hybridization of 500 ng oligo dT primers to 4 μg RNA at 65 °C for 5 min followed by extension at 42 °C for 50 min in the presence of 200 U Superscript II reverse transcriptase (Invitrogen, Brondby, Denmark), 500 μM dNTP, 10 μM DTT, 50 mM Tris–HCl, 75 mM KCl, and 3 mM MgCl_2_, pH 8.3. Finally, the RT was inactivated at 70 °C for 15 min. PCR was performed in a total volume of 20 μL containing 1 μL of the RT reaction, 0.5 mM dNTPs, 0.5 μM of each primer, 2 mM MgCl_2_ and 2 U Taq polymerase in PCR buffer (conventional PCR) or 2 U Pfu Ultra II fusion HS polymerase (Stratagene, Santa Clara, CA, USA) in Pfu PCR buffer (for cloning). PCR consisted of a denaturizing step of 2 min at 95 °C followed by 35 cycles of 95 °C in 30 s, 58 °C in 30 s and 72 °C in 2 min. Primers used for PCR were: Ano1_for; 5′-gacctgggctat gaggttca-3′, Ano1_rev; 5′-ggctgatgtctttggggata-3′, Ano6_for; 5′-gcagcccttggatcttatca-3′, Ano6_rev; 5′-tgctgtagctcaacggtg tc-3′, Ano10_for; 5′-tctgagtggaccagccttct-3′, Ano10_rev; 5′-agaagagtgaggcgaagcaa-3′. Primers used to clone and generate attB-flanked gateway compatible PCR products were Ano6_for; 5′-ggggacaagtttgtacaaaaaagcaggcttcaccatgcagatgatgactaggaaggtc-3′, Ano6_rev; 5′-ggggaccactttgtacaagaaagctgggtctcattcgagttttggccgcacg-3′, Ano1_for; 5′-ggggacaagtttgtacaaaaaagcaggcttcaccatgagggtccccgagaagtac-3′, Ano1_rev; 5′-ggggaccactttgtacaagaaagctgggtcctacagcgcgtccccatggga-3′, Ano10_for; 5′-ggggacaagtttgtacaaaaaagcaggcttcaccatgagagtgactttatcaacgctg-3′, Ano10_rev; 5′-ggggaccactttgtacaagaaagctgggtctcaggtagcttccttcccatct-3′. PCR products were excised from 0.8 % agarose gels and DNA extracted using E.Z.N.A gel extraction kit (Omega Bio-tek, Norcross, GA, USA). PCR products of m*Ano6*, m*Ano1* or m*Ano10* were then recombined into pDONR221 using BP Clonase according to the manufacturer’s instructions (Invitrogen, Taastrup, Denmark) to generate m*Ano6*, m*Ano1* or m*Ano10* entry clones. After transformation into E. coli and selection using 50 μg/mL kanamycin, DNA was purified and inserts in entry clones confirmed by full-length DNA sequencing. Finally, using LR-clonase mix, the inserts from entry clones were recombined into pcDNA6.2/EmGFP-Bsd/V5-DEST and pcDNA3.1-DEST47 to generate vectors co-expressing mANO6, mANO1 or mANO10 and EmGFP. Expression plasmids were produced in E. coli using ampicillin (100 μg/mL) as selection agent.

To determine the m*Ano6* mRNA level in EATC (WT and KD), real-time qPCR was performed in triplicates (EATC) using a Stratagene MX4000 Real-Time PCR system and SYBRGreen PCR Master Mix (ABI) in a total volume of 20 μL containing 1 μL of the RT reaction, 200 nM of primers and 10 μL 2× mastermix. Primers used for qPCR were: Ano6_for; 5′-gcagcccttggatcttatca-3′, Ano6_rev; 5′-tgctgtagctcaacggtg tc-3′, Arp_for; 5′-cgacctggaagtccaactac-3′, Arp-rev; and 5′-atctgcatctgcttg-3′. Target expression level was normalized to the reference gene level (m*Arp*) and the relative expression ratio calculated using the equation:

$$ {E}_{\mathrm{ratio}}=\frac{E_{\mathrm{target}}{{}^{\varDelta C{(t)}_{\mathrm{target}}}}^{\left(\mathrm{control}\div treated\right)}}{{E_{\mathrm{ref}}}^{\varDelta C{(t)}_{\mathrm{ref}}\left(\mathrm{control}\div treated\right)}} $$

where *E*_target_ and *E*_ref_ are the PCR amplification efficiencies for the target gene and reference gene, respectively, and Δ*C*(*t*)_target_ and Δ*C*(*t*)_ref_ the change in C(t) values for target and reference gene. ANO6 and ANO1 mRNA and protein levels in WT and KD ELA cells were previously determined by Jacobsen et al. [13].

### Gel electrophoresis and Western blotting

Cells were lysed in 95 °C lysis buffer (150 mM NaCl, 20 mM HEPES, 1 mM EDTA, 10 % Glycerol, 0.5 % Triton X-100, and 0.5 % SDS) with protease inhibitors (Roche Applied Science) and phosphatase inhibitors added. The lysates were subsequently homogenized, and the supernatant was collected by centrifugation. Protein concentrations were determined using Bio-Rad DC protein assay. SDS-polyacrylamide gel electrophoresis were performed using a 10 % gel (Invitrogen) and transferred to nitrocellulose membranes. The membranes were dyed with Ponceau Red (Sigma) and washed in TBST (10 mM Tris · HCl, pH 7.5, 120 mM NaCl, and 0.1 % Tween 20) before being blocked in a 5 % nonfat dry milk and TBST solution. Membranes were incubated with primary antibody at following concentrations: 1:50 ANO6 (gift from Prof. Kunzelmann), 1:8000 Alpha-tubulin. Membranes were washed in TBST before introduction to secondary antibody (Sigma) 1:5000. The protein bands were visualized using 5-bromo-4-chloro-3-indolyl phosphate-nitro blue tetrazolium (BCIP/NBT; Kirkegaard and Perry Lab) and scanned or alternatively visualized using using enhanced chemiluminiscence using horseradish peroxidase and luminol.

### Construction of microRNA plasmids

Interfering microRNA (miRNAi) KD of mANO6 and mANO1 was achieved using BLOCK-iT™ Pol II miR RNAi Expression technology (Invitrogen, Taastrup, Denmark). miRNAi targeting m*Ano6* and m*Ano1* were designed using Invitrogen’s online design tool generating sense and antisense single-stranded DNA strings (ssDNA). DNA sequences were: ANO6, sense, 5′-tgctgtttagcgggagtttgatgtgcgttttggccactgactgacgcacatcactcccgctaaa-3′, antisense, 5′-cctgtttagcgggagtgatgtgcgtcagtcagtggccaaaacgcacatcaaactcccgctaaac-3′; ANO1, sense, 5′-gacctgggctat gaggttca-3′ and antisense 5′-ggctgatgtctttggggata-3′. The two ssDNAs were annealed in annealing buffer generating a dsDNA, which was then ligated into pcDNA™6.2-GW/EmGFP-miR generating miR-ANO6-KD or miR-ANO1-KD plasmids. Plasmids were transformed into omnimax T-1 E. coli (Invitrogen, Taastrup, Denmark) and selected using spectinomycin as antibiotic (50 μg/μL). Plasmid inserts were confirmed by sequencing.

### Generation of mANO6 or mANO1 stable KD in EATC and ELA

EATC and ELA cells were transfected with miR-ANO6-KD or miR-ANO1-KD plasmids using Lipofectamine 2000 (Invitrogen, Taastrup, Denmark), incubated for 4 h and then re-suspended in fresh medium containing 10 μg/mL blasticidin. After 1 week selection, single cells were picked and transferred to 24- or 96-well trays and allowed to grow into a full clone in the presence of blasticidin. Clones were analyzed using qPCR (as described above) and, if possible, using Western blot analysis [13]. The selected EATC KD clone was named miR-ANO6-1 (EATC ANO6-KD) and the selected ELA KD clones were named miR-ANO6-10 (ELA ANO6-KD), miR-ANO6-15 and miR-ANO1-7 (ELA ANO1-KD).

### Electrophysiological recordings

Cells were plated on poly-l-lysine-coated cover slips. In experiments with overexpression of ANO proteins, transfected cells were identified by their EmGFP expression using a fluorescence microscope. Whole-cell voltage-clamp recordings were performed with the Axopatch 200B amplifier interfaced to a Digidata 1440A using pClamp10 for recording and analysis (Molecular Devices). Analog signals were acquired at 2.5 kHz and filtered at 1 kHz. All recordings were performed at room temperature (20 °C). Patch pipettes were fabricated from borosilicate glass capillaries using a DMZ-Universal Puller (Zeitz Instruments, Munich, Germany) with a resistance of 2–5 MΩ when filled with the internal solution. For activation of *I*_Cl, swell_, the pipette solution contained (in mM): CsCl, 90; MgCl_2_, 2; EGTA, 10; HEPES, 10; Na_2_-ATP, 5, pH 7.2 with TRIS or NaOH and osmolality adjusted to 295 mOsmol/kg with D-mannitol. The bath was a modified Ringer’s solution and, in the EATC and HEK293 experiments, it contained (in mM): NaCl, 90; CaCl_2_, 1; MgCl_2_, 1; and HEPES, 10, at pH 7.4 with TRIS and osmolality adjusted to 190 mOsmol/kg (hypotonic) or 300 mOsmol/kg (isotonic) with d-mannitol. For ELA experiments, the bath contained (in mM): NaCl, 150; CaCl_2_, 1.5; MgCl_2_, 1; HEPES, 10; glucose, 10, pH 7.4 with NaOH and osmolality adjusted to 317 mOsmol/kg (isotonic) with d-mannitol. To produce the hypotonic bath, the isotonic solution was diluted 30 % (final osmolality: 222 mOsmol/kg). Osmolality was measured using a vapor pressure depression osmometer (Wescor Vapro 5520). Series resistance was compensated by 60–70 %.

Liquid junction potential changes due to extracellular Cl^−^ replacement were calculated with the Clampex10 software (Axon Instruments) and corrected for by *V*_m_ = *V*_test_ − 3.8 mV (EATC and HEK293 experiments) or *V*_m_ = *V*_test_ − 4.2 mV (ELA experiments) for *I*_Cl, swell_. A ramp protocol ranging from *V*_test_ = −100 to +100 mV (EATC experiments) or *V*_test_ = −80 to +80 mV (ELA experiments) over 1 s with a holding potential of *V*_test_ = 0 mV was run continuously with 15 s intervals to follow the current activation. A step protocol with a holding potential, *V*_hold_ = 0 mV and steps of 1 s duration ranging from *V*_test_ = −100 to +100 mV with 20 mV increments was applied before and after hypotonic stimulation of *I*_Cl, swell_.

### Cell volume measurements

Cell volume was measured by electronic cell sizing in a Beckman–Coulter Multisizer™ 3 (Beckman–Coulter, Copenhagen, Denmark). The tube orifice was 100 μm. Cell density was 20,000 cells/mL, equivalent to a cytocrit of 0.002 %. Mean cell volume was calculated as the median of the volume distribution curves after calibration with latex beads (nominal diameter 10 μm). Volume measurements were carried out in 37 °C isotonic Ringer’s solution (274 mOsmol/kg) for initial cell sizing of the control volume, and in 37 °C hypotonic Ringer’s solution (160 mOsmol/kg) for the RVD responses. Media used for cell volume measurements were filtered (Millipore filters, 0.45 mm) prior to experiments. Both Ringer’s solutions contained (in mM): Na^+^, 75; Cl^−^, 75; K^+^, 2.5; Mg^2+^, 0.5; Ca^2+^, 0.5; SO_4_^2−^, 0.5; HPO_4_^2–^, 0.5; MOPS, 3.3; TES, 3.3; HEPES, 5. The osmolality of the isotonic solution was adjusted using sucrose. For Ca^2+^-free experiments, CaCl_2_ was substituted by NaCl and EGTA was added to a concentration of 1 mM.

### Taurine efflux measurements

ELA WT and KD cells were grown in 6-well plates to 80 % confluency. Before the start of the experiment, the cells were loaded with ^3^H-taurine (final concentration: 0.04 μM) and incubated for 2 h at 37 °C and 5 % CO_2_. After incubation, the cells were washed with an isotonic (300 mOsmol/kg) NaCl solution containing (in mM): NaCl, 143; KCl, 5; Na_2_HPO_4_, 1; CaCl_2_, 1; MgSO_4_, 1; HEPES, 10, and pH adjusted to 7.4. A volume of 1 mL of isotonic NaCl was added to each well at time zero, and every 2 min the solution in each well was transferred to a vial and replaced by either 1 mL fresh isotonic solution (0–8 min) or fresh hypotonic (200 mOsmol/kg) solution (10–24/30 min). The hypotonic solution was obtained by dilution of the isotonic solution with buffered water. Following the final removal of hypotonic solution, 1 mL of 1 M NaOH was added to each well to lyse the cells. The six-well plate was placed on a shaking table for a minimum of 1 h. The cell lysates were then transferred to vials; 1 mL ddH_2_O was added to each well and subsequently transferred to a vial (this was repeated twice); 3.5 mL Ultima Gold™ scintillation cocktail (PerkinElmer) was added to each vial and the vial was shaken thoroughly. ^3^H activity was measured using a beta-scintillation counter (Packard). Assuming that there is only one channel type facilitating the taurine release, and that taurine release follows a mono-exponential time course, the fractional rate constant (*k*) for taurine release in each time interval can be deduced from the following equation: *A*_*t*_ = *A*_*t*–Δt_*e^−k*Δ*t*^, where *A*_*t*_ is the cellular activity of ^3^H-labeled taurine at the end of the given time interval (counts per minute), *A*_*t*–Δ*t*_ is the activity at the beginning of the interval (counts per min), *k* is the fractional rate constant (minutes), and Δ*t* is the length of the interval (2 min). Rate constants for taurine release under isotonic conditions were estimated as the average of release rates at *t* = 8 min and *t* = 10 min, whereas the values for hypotonic conditions were estimated as the maximal transport rate after shift to hypotonic bath solution.

### Caspase-3 activity assay

Caspase-3 activity was measured using the Fluorometric Homogenous Caspases Assay (Roche Applied Science). 10,000 ELA WT or KD cells per well were added to two 96-well plates. The two 96-well plates were identical and used for caspase-3 activity measurements and protein determination, respectively. The plates were incubated at 37 °C and 5 % CO_2_ for 24 h. The medium was then replaced by medium containing 10 μM cisplatin and the plates were incubated at 37 °C and 5 % CO_2_ for 24 h. In the plate used for protein determination, the cells were washed in PBS, and lysis buffer (10 mM Tris-Cl, 1 % SDS) was added. This plate was then incubated at -80 °C for 24 h and protein concentrations were determined using a colorimetric DC protein assay (Bio-Rad), a FLUOstar Optima plate reader (BMG Labtech), and a standard curve based on samples with known protein concentrations. The plate used for caspase-3 activity measurements was frozen (30 min at −80 °C) and thawed (45 min at RT) three times to promote cell lysis. DEVD-R110 and incubation buffer from the assay kit was added to the cells and the plate was covered in aluminum foil and incubated at 37 °C for 1 h. Fluorescence was then measured at 520 nm with an excitation wavelength of 485 nm using the FLUOstar Optima plate reader. The caspase-3 activity of each cell line was calculated as the mean of the measurements in either three or five wells and fluorescence from wells containing only growth medium, DEVD-R110 and incubation buffer was subtracted from the measurements to remove background fluorescence. The caspase-3 activities were then normalized to the measured protein concentrations. Finally, all results were normalized to the caspase-3 activity of WT cells that had not been exposed to cisplatin.

### Statistics

ANO-KD or ANO-expressing cells were compared with WT or mock-transfected cells using two-sided Student’s *t* tests assuming either equal or unequal variances depending on the result of an *F* test. The results were considered significant when *p* < 0.05.

## Results

### *I*_Cl, swell_ in mANO1, mANO6, or mANO10-transfected HEK293 cells and mANO6-transfected EATC

**Fig. 1:**
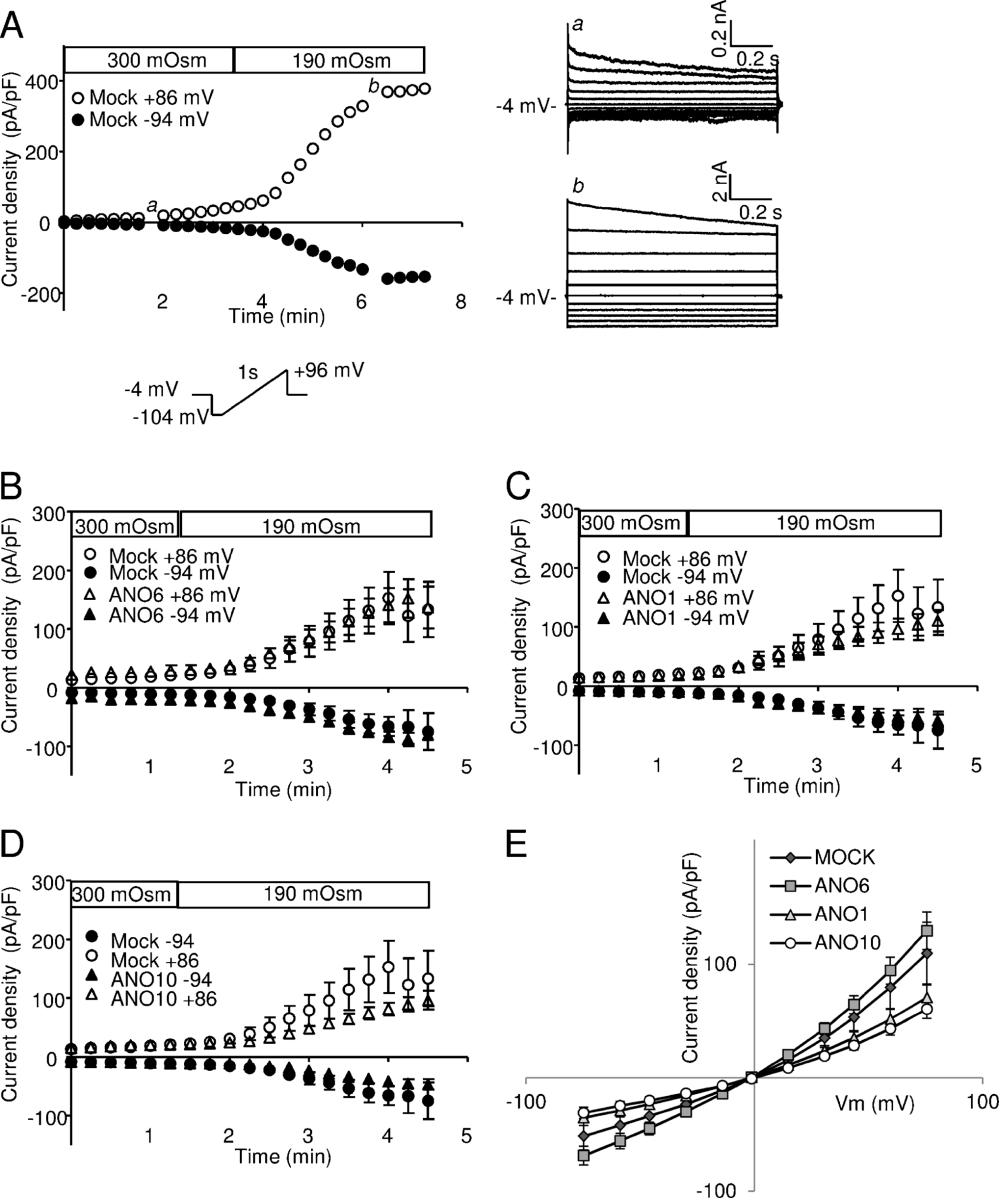
VRAC currents in mANO1, mANO6, and mANO10 transfected HEK293 cells. Measurements were conducted with no intracellular Ca^2+^ and 10 mM EGTA. **a** Representative current development (*left*) and current–voltage dependency (*right*) of the endogenous VRAC current in a mock-transfected HEK293 cell exposed to hypotonic superfusate. **b** VRAC current development in mANO6 (*n* = 5), **c** mANO1 (*n* = 9), and **d** mANO10 (*n* = 11) transfected HEK293 cells; each compared with mock (*n* = 8). **e** The average IV-relations at maximal current for mock and mANO6-, mANO1-, and mANO10-transfected cells, generated from ramp protocol sweeps (from −104 to +96 mV). Data in (**b**–**e**) are represented as mean ± SE and in (**e**), WT was compared with the ANO-transfected cells at ±15, ±30, ±45, ±60, and ±75 mV using two-sided Student’s *t* tests and no significant differences were found

To investigate whether anoctamins generate volume-sensitive anion currents, we measured the swelling-activated whole cell Cl^−^ current (*I*_Cl, swell_) under strongly Ca^2+^-buffered conditions (at a nominal (Ca^2+^)_p_ of zero and 10 mM EGTA in the pipette solution, i.e., no contribution from CaCC). Under these conditions, perfusion with a 36 % hypotonic solution resulted in visible cell swelling and development of an outwardly rectifying current (Fig. 1a). This membrane current showed all the classical features typical of VRAC as described for many cell types [12, 25], e.g., time-dependent inactivation following voltage-clamp steps to high positive potentials (Fig. 1a, right). The deactivation at positive potentials is in contrast to what has been found for the ANO6 channel (compare with [9], Figs. 3 and 4). The swelling-activated currents were recorded in mock-, mANO1-, mANO6-, and mANO10-expressing HEK293 cells, which were visually identified by their EmGFP expression. Figure 1b–d shows the average time courses of the current development toward steady state following hypotonic treatments. Although we expected to see an increase in current if ANO6 contributed to the swelling-activated current, no significant changes in the current was found in either mANO1-, mANO6-, or mANO10-expressing cells when compared with mock-transfected cells (Fig. 1e). If anything, there was a slight tendency that ANO1 and ANO10 overexpression induced a suppression of the endogenous volume-activated current (Fig. 1c–e). It should be noted that the present patch clamp experiments were performed at room temperature, but even though ANO1 is shown to be temperature dependent in nociceptive neurons [3], the contribution of both ANO1 and ANO6 to the total Ca^2+^-activated current is still prominent in the transfected HEK cells at room temperature [9]. The reversal potentials measured at 180 s of hypotonic stimulation were also unchanged. Expression of mANO6 in EATC cells did not significantly change the volume-activated current compared with WT either (maximal VRAC current at −75 mV: WT, −23.1 ± 4.7 p*A*/p*F* (*n* = 16); mANO6 overexpression, −20.6 ± 4.0 pA/pF (*n* = 6), *p* = 0.76). Thus, overexpression of neither of the studied anoctamins enhanced the volume-sensitive currents of HEK293 cells or EATC under conditions of zero intracellular calcium.

### *I*_Cl, swell_ in EATC and ELA cells with stable KD of ANO6

**Fig. 2:**
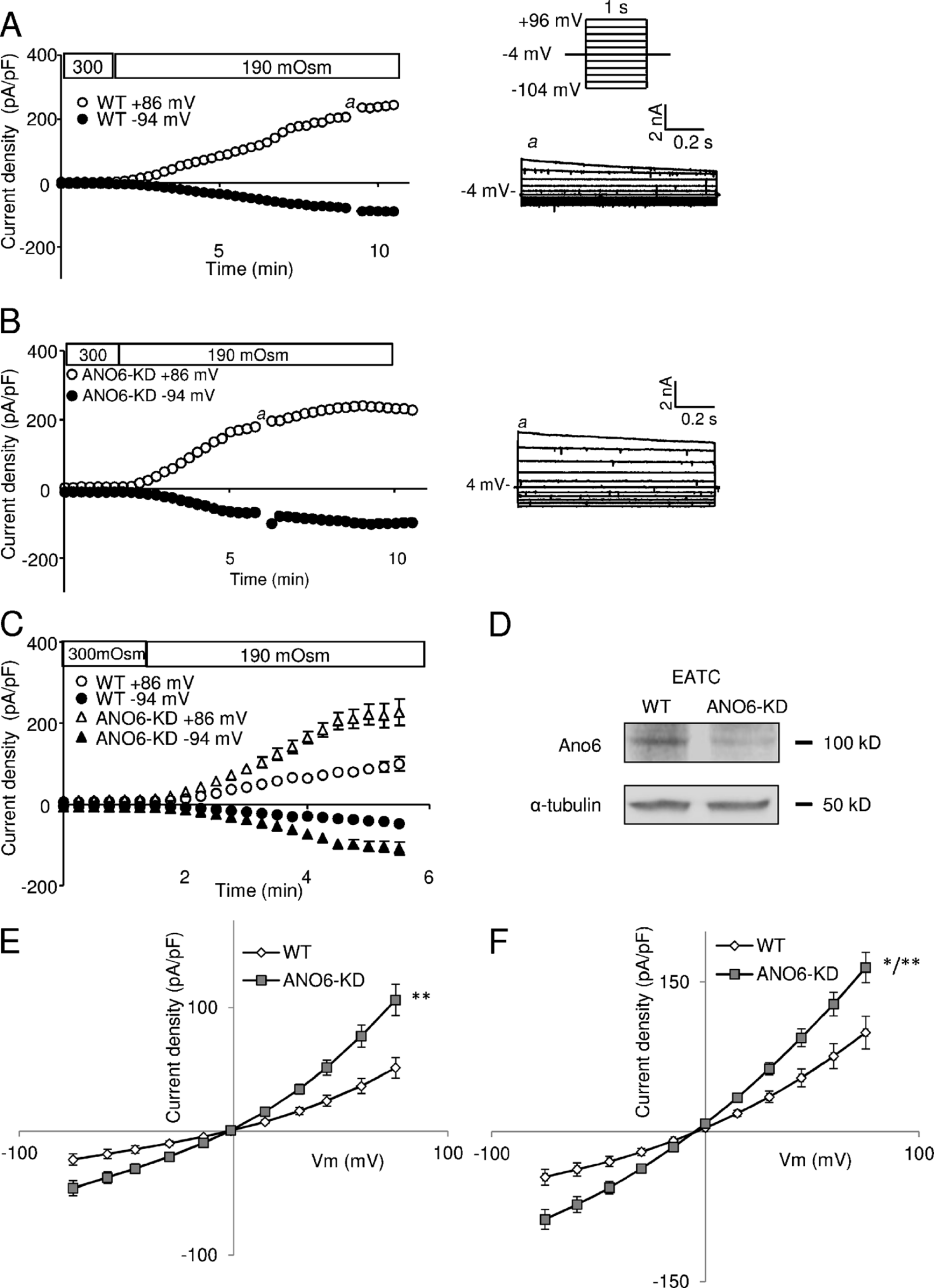
VRAC currents in WT and ANO6-KD EATC and ELA cells. Measurements were conducted with no intracellular Ca^2+^ and 10 mM EGTA. **a** Representative current development (*left*) and current–voltage dependency (*right*) of the endogenous VRAC current in a WT EATC exposed to hypotonic solution. **b** As in (**a**) with an ANO6-KD cell. **c** VRAC current development in WT (*n* = 15) and ANO6-KD (*n* = 23) EATC exposed to hypotonic solution. **d** Western blot demonstrating KD of ANO6 in EATC. Blot is typical of three separate experiments. **e** WT and ANO6-KD EATC: the average IV relation at maximal current generated from ramp protocol sweeps (from −104 to +96 mV). **f** WT and ANO6-KD ELA cells: the average IV relation at maximal current generated from ramp protocol sweeps (from −84 to +76 mV). Values in (**c**), (**e**), and (**f**) represent mean ± SE and in (**e**) and (**f**), WT was tested against KD at ±15, ±30, ±45, ±60, and ±75 mV using two-sided Student’s *t* tests (e; **p < 0.01 at all points; f; *p < 0.05; **p < 0.01)

To further investigate the relationship between ANO6 and the swelling-induced anion current, we compared whole cell currents under hypotonic and Ca^2+^-free conditions in WT and two different ANO6 KD clones, in EATC and ELA cells, respectively. KD in EATC (KD clone: miR-ANO6-1) was confirmed using qPCR, which showed a significant 45 ± 11 % reduction (*p* = 0.009; *n* = 6) in the ANO6 mRNA level compared with WT, and Western blotting (Fig. 2d). KD in ELA cells (KD clone: miR-ANO6-10) on both mRNA and protein levels was previously shown by Jacobsen et al. (see [13], Fig. 1a). KD of ANO6 in ELA cells was not compensated by a concomitant upregulation of ANO1 and ANO10 [13]. From Fig. 2a, b, it is seen that the current was activated shortly after application of hypotonic extracellular solution and had the properties characteristic of VRAC. If ANO6 contributed to the swelling-activated current, we would expect the ANO6-KD to decrease the current. Instead, we found that ANO6-KD in both EATC (Fig. 2c, e) and ELA cells (Fig. 2f) resulted in a significant increase in VRAC activation. This surprising result may indicate interaction between the expression levels of the two anion channels, the mechanism of which is unknown. What is of importance for our argumentation, however, is that the volume-activated currents are not decreased in ANO6-KD cells.

### RVD in WT and ANO6-KD EATC

**Fig. 3:**
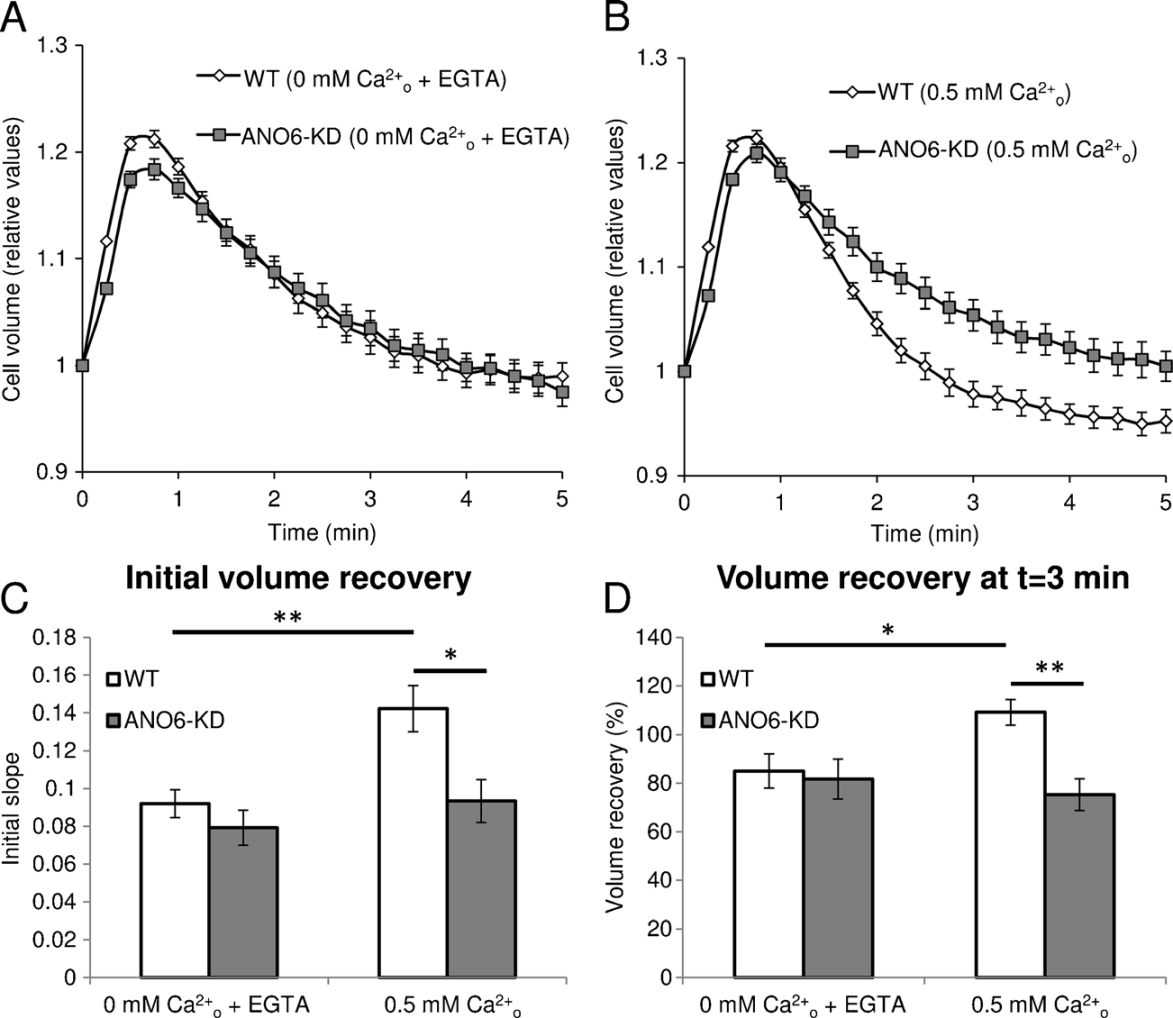
Cell volume regulation in WT and ANO6-KD EATC. **a** Cell volume measured by electronic cell sizing (Coulter Counter) over a 5-min period following exposure to a 42 % hypotonic medium containing no Ca^2+^ and 1 mM EGTA. Values are given relative to the initial cell volume of each cell line and represent mean ± SE of seven experiments. **b** Cell volume measured as in (**a**) with EGTA in the medium replaced by 0.5 mM Ca^2+^. Values represent mean ± SE of 8 and 14 experiments in WT and ANO6-KD EATC, respectively. **c** The absolute value of the initial slope of the volume recovery from the time of maximal volume to 1 min after is based on the values in (**a**) and (**b**). Values are presented as mean ± SE. Using two-sided Student’s *t* tests, WT and ANO6-KD were compared with each other (0 mM Ca^2+^_o_ + EGTA, *p* = 0.30; 0.5 mM Ca^2+^_o_, *p* = 0.012) and WT without Ca^2+^ was compared with WT with Ca^2+^ (*p* = 0.005). **d** The 3-min volume recovery was determined from maximal volume towards the original volume after 3 min of hypotonic exposure, based on the values in (**a**) and (**b**). Values are represented as mean ± SE, and, using two-sided Student’s *t* tests, WT and ANO6-KD were compared with each other (0 mM Ca^2+^_o_ + EGTA, *p* = 0.76; 0.5 mM Ca^2+^_o_, *p* = 0.002) and WT without Ca^2+^ was compared with WT with Ca^2+^ (*p* = 0.015). *p < 0.05; **p < 0.01

To explore if ANO6, like VRAC, has a Ca^2+^-independent role in cell volume regulation, we measured RVD, i.e., the swelling-induced cell volume recovery, in WT and ANO6-KD EATC with extracellular Ca^2+^ replaced by 1 mM EGTA. Under such conditions, plasma membrane Ca^2+^ influx is eliminated without affecting Ca^2+^ release from intracellular stores. As the volume-sensitive K^+^ and Cl^−^ fluxes, and hence RVD, are temperature dependent [30], we performed the Coulter counter experiments at 37 °C to evaluate the effect of ANO6-KD under more physiological conditions. KD of ANO6 (KD clone: miR-ANO6-1) was confirmed as described above. As seen in Fig. 3a, exposure of WT EATC to a 42 % hypotonic shock induces rapid cell swelling which is followed by the classic RVD response as described previously [12]. The extent of the RVD response was calculated in two ways: as the absolute value of the initial slope of volume recovery from the time of maximal volume to 1 min after (Fig. 3c) and as the recovery from maximal cell volume toward the original volume after 3 min of hypotonic exposure (Fig. 3d). The initial slope in the WT cells was 0.092 ± 0.007 min^−1^ (*n* = 7) and was not significantly different from the slope in ANO6-KD cells, which was 0.079 ± 0.009 min^−1^ (*n* = 7; *p* = 0.30) (Fig. 3c). Similarly, the recovery after 3 min in the WT and ANO6-KD cells was 85 ± 7 % (*n* = 7) and 82 ± 8 % (*n* = 7), respectively, and no significant difference was seen between the two (*p* = 0.76) (Fig. 3d). Thus, these data show that ANO6, unlike VRAC, is not able to contribute to RVD in the absence of extracellular Ca^2+^. From Figs. 2e and 3a, it is seen that VRAC activity in EATC is higher in ANO6-KD compared with WT cells, whereas both cell lines show similar RVD efficiency. This conforms with the previous demonstration that the K^+^ conductance is rate limiting in the RVD response in EATC, which prevents an increase in the Cl^−^ conductance to be seen [12].

We then tested if ANO6, through its role as a CaCC, could contribute to RVD when extracellular Ca^2+^ was present. In this experiment, the ANO6-KD cells showed a significantly lower initial slope of 0.093 ± 0.01 min^−1^ (*n* = 14) when compared with 0.14 ± 0.01 min^−1^ (*n* = 8) in the WT cells (*p* = 0.012) (Fig. 3c). The recovery after 3 min in the ANO6-KD cells, 75.3 ± 6.5 % (*n* = 14), was also significantly lower compared with WT, 109.2 ± 5.2 % (*n* = 8; *p* = 0.002) (Fig. 3d). When looking at WT cells, the data show that removal of extracellular Ca^2+^ significantly reduces both the initial slope (*p* = 0.005) (Fig. 3c) and the recovery after 3 min (*p* = 0.015) (Fig. 3d), whereas removal of extracellular Ca^2+^ from ANO6-KD cells had no significant effect on neither the initial slope (*p* = 0.43) nor the recovery after 3 min (*p* = 0.56). These results suggest that Ca^2+^ plays an important role in RVD in EATC, and that this is, at least partly, due to the Ca^2+^-dependent activity of ANO6. This seems to contradict previous results from our group which showed that RVD in EATC does not depend on Ca^2+^ [14]. However, the EATC investigated in our previous study was cultured in the abdominal cavity of female Naval Medical Research Institute mice, whereas EATC in the present study were cultured in RPMI-1640 medium. The most likely explanation is that the in vivo-generated cell line had a lower level of anoctamin expression. However, as this cell line is no longer available, a direct proof of this notion cannot be carried out.

### Taurine efflux in WT and ANO1- and ANO16-KD ELA cells

**Fig. 4:**
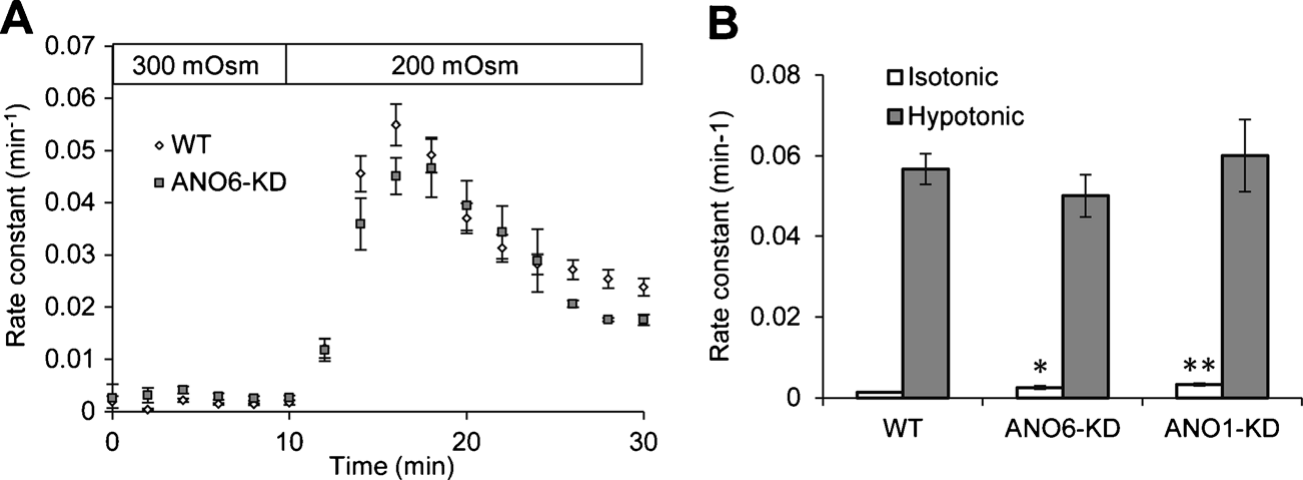
Taurine efflux in WT and ANO1- and -6-KD ELA cells. **a** Taurine efflux in WT (*n* = 24) and ANO6-KD (*n* = 7) ELA cells was measured for 30 min using the radiotracer technique and the efflux rate constants were estimated in each 2-min time interval. After 10 min in isotonic solution, the cells were exposed to a 33 % hypotonic solution. **b** Taurine efflux was measured as in (**a**) in WT (*n* = 24), ANO6-KD (*n* = 7), and ANO1-KD (*n* = 9) ELA cells. The isotonic rate constant was estimated as the average of rate constants at *t* = 8 min and *t* = 10 min, whereas the hypotonic rate constant was estimated as the maximal rate constant after hypotonic exposure. Data in (**a**) and (**b**) are represented as mean ± SE and in (**b**), WT was compared with ANO-KDs using a two-sided Student’s *t* test (ANO6-KD, *p* = 0.038 (isotonic); *p* = 0.39 (hypotonic); ANO1-KD, *p* = 0.000 (isotonic); *p* = 0.69 (hypotonic)). *p < 0.05; **p < 0.01

Efflux of organic osmolytes, such as taurine, plays an important role in RVD [12, 20]. Despite this, the molecular identity of the VSOAC, which facilitates this efflux, is not known. Here, we investigate whether ANO1 and ANO6 could contribute to the volume-sensitive release of taurine. Taurine efflux in WT and ANO-KD ELA cells was measured using the radiotracer efflux technique during isotonic and hypotonic conditions. KD of ANO6 and ANO1 was confirmed by Jacobsen et al. [13]. In both WT and ANO-KD cells, exposure to hypotonic solution resulted in a fast increase in the taurine efflux rate constants followed by a slow decrease (Fig. 4a). In contrast to what would be expected if anoctamins contributed to taurine efflux, the taurine efflux rate constants in ANO1- and ANO16-KD cells were similar to or higher than those of WT cells (Fig. 4b). The ANO6-KD clone had a significantly higher efflux than WT cells at isotonic conditions (*p* = 0.038), while there was no significant difference at hypotonic conditions (*p* = 0.39). In the ANO1-KD clone, taurine release at isotonic conditions was significantly higher than in WT cells (*p* = <0.001), but not significantly higher under hypotonic conditions. The results of this functional test indicate that neither ANO1 nor ANO6 are VSOAC.

### Cisplatin-induced caspase-3 activity in WT and ANO6-KD ELA cells

**Fig. 5:**
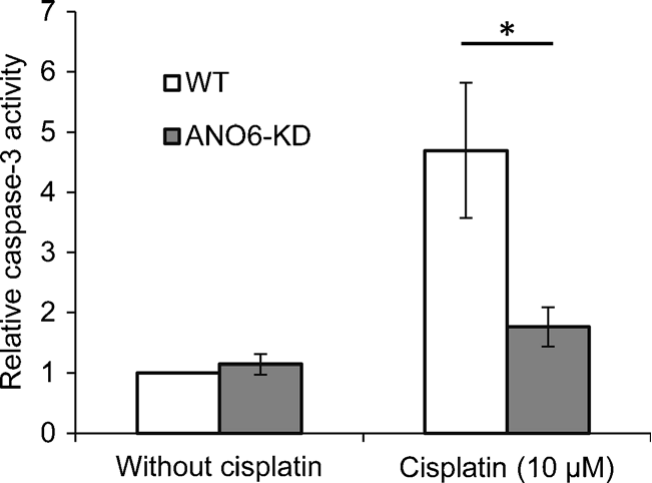
Caspase-3 activity in WT and ANO6-KD ELA cells. Caspase-3 activity in WT (*n* = 11) and ANO6-KD (*n* = 11) ELA cells was measured after 24 h exposure to medium containing either no or 10 μM cisplatin using a fluorometric assay, and values were then normalized to the caspase-3 activity of WT cells without cisplatin. Data are represented as mean ± SE and WT was compared with ANO6-KD using a two-sided Student’s *t* test (*p* = 0.41 (without cisplatin), *p* = 0.028 (10 μM cisplatin)). *p < 0.05

Apoptotic volume decrease (AVD) is an essential early step in the apoptotic process [15, 22] and several ion channels involved in RVD are also involved in AVD [1, 21, 26]. As ANO6, in the presence of extracellular Ca^2+^, is involved in RVD, we investigated if KD of ANO6 affected the cells’ ability to undergo apoptosis. Using a fluorometric assay, we measured the caspase-3 activity in WT and ANO6-KD ELA cells, which either had or had not been exposed to the apoptosis-inducing drug cisplatin for 24 h (Fig. 5; results from KD clone miR-ANO6-10 are shown; values are relative to the caspase-3 activity of WT cells not exposed to cisplatin). Without cisplatin, no significant difference was seen between WT and KD cells (WT, 1.0 (*n* = 11); miR-ANO6-10, 1.14 ± 0.17 (*n* = 11; *p* = 0.41); and miR-ANO6-15, 0.74 ± 0.21 (*n* = 4; *p* = 0.30)). However, following cisplatin exposure, ANO6-KD cells showed significantly less caspase-3 activity compared with WT cells (WT, 4.70 ± 1.1 (*n* = 11); miR-ANO6-10, 1.76 ± 0.34 (*n* = 11; *p* = 0.028); and miR-ANO6-15, 1.08 ± 0.07 (*n* = 4; *p* = 0.0092)). These results suggest that ANO6 is involved in cisplatin-induced apoptosis.

## Discussion

### ANO6 and the volume-activated anion current

It has previously been suggested that ANO6 is activated following cell swelling [1], and it could therefore be speculated that ANO6’s activity is volume regulated. In the present study, however, expression of mANO1, mANO6, or mANO10 in HEK293 cells and mANO6 in EATC, did not affect swelling-activated currents in the absence of intracellular Ca^2+^ (10 mM EGTA added to the pipette solution) (Fig. 1e). Moreover, KD of ANO6 in EATC and in ELA cells did not reduce the swelling-induced current (Fig. 2e, f). These results are showing that ANO6 as well as ANO1 and ANO10 do not contribute to cell volume-activated currents, which is in agreement with the results obtained by Shimizu et al. [31]. This is not in conflict with the study by Almaca et al. who found that expression of ANO1 and ANO2 enhanced *I*_Cl, swell_ in whole-cell patch clamp experiments with 0.1 μM-free Ca^2+^ in the pipette, and knock-down of ANO1, ANO6, ANO8, and ANO9 reduced *I*_Cl, swell_ under the same conditions [1]. They hypothesized that the anoctamin-mediated *I*_Cl, swell_ was stimulated through an autocrine mechanism that involves swelling-induced ATP release, binding to the purinergic P2Y_2_ receptors with a subsequent increase in intracellular Ca^2+^. It follows that anoctamins’ role in *I*_Cl, swell_ and RVD would be secondary to an increased intracellular (Ca^2+^).

### ANO6 is involved in RVD in a Ca^2+^-dependent manner

EATC are well suited for the study of the putative role of anoctamin channels in RVD. It is the first cell type in which an increased Cl^−^ permeability following hypotonic cell swelling was demonstrated [10], and the cell swelling-activated current as well as Ca^2+^-activated current are biophysically and pharmacologically well characterized in these cells [27]. In a variety of cells, VRAC as well as CaCC are known to be associated with the RVD response [12]. However, VRAC in ELA cells does not require Ca^2+^ for activation [16] (see also Figs. 1 and 2), whereas CaCCs are dependent on influx of Ca^2+^. To approach the question of whether ANO6 is swelling-activated per se, we investigated if ANO6 has a Ca^2+^-independent role in RVD by studying cell volume recovery in WT and ANO6-KD EATC in the absence of extracellular Ca^2+^. Our results show that there is no significant difference between WT and ANO6-KD cells in their ability to undergo RVD in Ca^2+^-free solution. Almaca et al. previously showed that ANO1, ANO6, ANO8, and ANO9 are essential for RVD in HEK293 cells and that *I*_Cl, swell_ is reduced in the colonic epithelium and in salivary acinar cells from mice lacking expression of ANO1 [1, 18]. However, these experiments were conducted in the presence of extracellular Ca^2+^. Thus, neither their results nor their hypothesis mentioned above contradict our conclusion. To replicate their results with our protocol, we repeated our experiment in the presence of extracellular Ca^2+^. We observed that ANO6-KD cells display the RVD response, but it proceeds at a significantly slower rate compared with the response in WT cells (Fig. 3c, d). This result shows that ANO6 contributes to the physiological RVD in EATC, but only in the presence of Ca^2+^, i.e., under normal physiological conditions. In whole cell patch clamp studies, the high intracellular Ca^2+^ required for ANO6 activation is secured by the free Ca^2+^ concentration in the pipette, whereas in the RVD response of intact cells, the high intracellular Ca^2+^ at the submembrane domains governing ANO6 activation depends on a putative Ca^2+^ influx during RVD. Apparently, in our experiments, this influx was sufficient for driving Ca^2+^ above the threshold for activation of ANO6. The effect of cell swelling on Ca^2+^_i_ differs widely between cell types, as does the dependence of RVD on extracellular Ca^2+^. This is summarized and discussed in [12]. The Ca^2+^ dependence of RVD is likely to reflect the involvement of Ca^2+^-activated K^+^ and/or Cl^−^ channels.

### ANO6 and ANO1 do not contribute to taurine efflux

Taurine is an essential organic osmolyte in most mammalian cells, and taurine release after cell swelling is an important component of the RVD response [12, 20]. Under isotonic conditions, taurine release is low and the mechanism behind it is unknown [20]. After hypotonic exposure, the VSOAC is activated, allowing taurine efflux to drastically increase [11, 12]. VSOAC has not been identified at the molecular level and was previously considered to be the same as VRAC. However, evidence now indicates that they are distinct channels [19]. As anoctamins are involved in RVD, we speculated if they were taurine channels. We therefore investigated the effects of ANO1- and ANO6-KD on taurine efflux under isotonic and hypotonic conditions. Our results clearly show that swelling-induced taurine release is not reduced by ANO-KD, i.e., ANO1 and ANO6 are different from VSOAC (Fig. 4b). In some KD clones, we observed a small but significant increase in taurine efflux compared with WT under isotonic conditions. This might be explained by a compensatory mechanism in which taurine release is increased to compensate for the lacking anoctamin activity.

### ANO6 is involved in cisplatin-induced apoptosis in ELA cells

AVD has been shown to be both necessary and sufficient to initiate apoptosis in some cell types [7, 8, 22]. AVD is very similar to RVD in many respects and several ion channels, including ANO1 and VRAC, are involved in both processes [1, 26, 29]. We therefore investigated if ANO6 is involved in apoptosis. Our results show that ANO6 plays an important role during cisplatin-induced apoptosis in ELA cells (Fig. 5). This is similar to what Martins et al. found in A549 cells [24], but in contradiction to the findings of Shimizu et al., who showed that KD of ANO6 does not affect staurosporine-induced AVD in HeLa cells [33]. However, a previous study from our group showed that the subset of ion channels activated following apoptotic stimuli can vary considerably between cell lines [35], and it is therefore quite possible that ANO6 only contributes to apoptosis in some cell types. With respect to RVD, we showed that influx of Ca^2+^ was required for ANO6 to play a role in RVD. It is likely that the same is true for AVD, i.e. only in cells where an early influx of Ca^2+^ is seen there will be a contribution of ANO6 to the AVD process.

## Conclusions

Our whole-cell patch clamp data demonstrate that ANO6-dependent Cl^−^ currents, unlike VRAC currents, are not activated by osmotic cell volume increase in the absence of Ca^2+^ in the pipette. However, there may be not-yet-described compensatory interactions between ANO6 and VRAC, as the latter tend to increase its activity in the absence of ANO6. Furthermore, we show that ANO6 plays a role in RVD provided there is Ca^2+^ in the bath, once again confirming the central role of Ca^2+^ in opening of ANO6 channels. Finally, we find that ANO6-KD reduces cisplatin-induced apoptosis in ELA cells, suggesting that ANO6 contributes to AVD.

## Glossary

AVD: Apoptotic volume decrease
CaCC: Calcium-activated chloride channel
EATC: Ehrlich ascites tumor cell
ELA: Ehrlich–Lettre ascites
HEK: Human embryonic kidney
KD: Knock-down
RVD: Regulatory volume decrease
VRAC: Volume-regulated anion channel
VSOAC: Volume-sensitive organic osmolyte and anion channel
WT: Wild type
